# A Special Issue on Fukushima Has Been Released a Decade After the Great East Japan Earthquake

**DOI:** 10.2188/jea.JE20220185

**Published:** 2022-12-05

**Authors:** Kotaro Ozasa, Kota Katanoda

**Affiliations:** 1 Health Management Center, Kyoto Prefectural University of Medicine, Kyoto, Japan; 2 Division of Surveillance and Policy Evaluation, National Cancer Center Institute for Cancer Control, Tokyo, Japan

**Keywords:** disasters, evacuation, mass screening, radiation

Here, we are publishing a special issue on the Fukushima Health Management Survey (FHMS). On March 11, 2011, the Great East Japan Earthquake and the Fukushima Daiichi Nuclear Power Plant accident occurred. As a consequence, enormous damage was inflicted on Fukushima Prefecture and other parts of Eastern Japan. The nuclear accident forced many residents around the power plant to evacuate, and even in 2022, 11 years after the accident, more than 30,000 residents are continuing to live as evacuees in and out of the prefecture.

The FHMS was initiated 3 months after the accident to investigate the effects of radiation exposure and to monitor and promote the long-term health of prefectural residents.^1^ The survey is the largest ever program of its kind, both nationally and internationally, on the health assessment and care of residents after a nuclear accident. It covers a wide range of health issues, comprising the following five surveys: the *Basic Survey*, to estimate external radiation doses; the *Thyroid Ultrasound Examination*, to monitor the thyroid gland status of children and provide long-term care for their health; the *Comprehensive Health Check*, to prevent, detect and treat lifestyle-related diseases at an early stage; the *Mental Health and Lifestyle Survey*, to assess the mental health and provide appropriate care; and the *Pregnancy and Birth Survey*, to assess the mental and physical health of expectant and nursing mothers, reduce their anxiety, and provide necessary care.

Regarding radiation exposure and health implications among residents, several reports have been published by international organizations. Among them, the United Nations Scientific Committee on the Effects of Atomic Radiation (UNSCEAR) released the 2013 Report^2^ and updated it in 2020 according to the newly available information.^3^ These two reports basically concluded that residents in Fukushima Prefecture, including evacuees, were estimated to be exposed to radiation externally and internally up to the levels of average effective doses of around ten millisieverts and average absorbed thyroid doses of several tens milligrays, although they varied among people. The reports indicated that no deterministic effects due to radiation exposure were documented among Fukushima residents and stochastic effects, including leukemia or any cancer related to radiation exposure, are unlikely to be discernible among them in the future. The excess detection of thyroid cancer among participants in the thyroid examination program was not thought to be the results of radiation exposure, but rather the results of sensitive ultrasound screening procedures. There was no evidence of excess congenital anomalies, stillbirths, preterm deliveries, or low birth weights among newborns related to radiation exposure. Observed increase in the incidence of cardiovascular and metabolic conditions was thought to be associated with concomitant social and lifestyle changes among residents, especially evacuees. Excess psychological distress was also thought to emerge in the aftermath of the combined disasters, including the nuclear power plant accident and subsequent changes in circumstances including evacuation. As for thyroid health monitoring after nuclear accidents, an expert group organized by the International Agency for Research on Cancer (IARC) recommended against population thyroid screening, but consideration to be given to offering a long-term thyroid monitoring program for higher risk individuals.^4^

In this special issue, major results of the FHMS for these 10 years after the accidents are introduced by the researchers themselves who have been involved in the survey. The first overview article summarizes the history, results, and future perspectives of the FHMS,^5^ followed by six review articles summarizing each of the above five survey components (the fifth and sixth review articles separately cover the *Pregnancy and Birth Survey*),^6^^–^^11^ and four original papers, which report the results of analyses using the *Thyroid Ultrasound Examination*,^12^ the *Comprehensive Health Check*,^13^ the *Mental Health and Lifestyle Survey*,^14^ and the *Pregnancy and Birth Survey*,^15^ respectively. The last three original papers used the external dose estimates updated in the review article in this issue.^6^

This special issue is intended to provide a forum for scientific discussion on the results of FHMS to date. It does not cover the issue of planning and its implementation of the FHMS itself. There has been much debate as to how the surveys should be continued, for example, the main body of the *Pregnancy and Birth Survey* was terminated at the end of the fiscal year 2020.^16^ However, such decisions require not only scientific evaluation but also social and ethical aspects and should be made by the authorities responsible for the implementation of the FHMS through consensus building with the relevant parties. The Editorial Board of the Journal of Epidemiology received a proposal for this special issue from a research group at Fukushima Medical University, which is involved in the FHMS. We decided to publish this issue because we thought it better to provide an opportunity for them to present how they summarize the current situation and future perspectives. We hope that this special issue will contribute to current and future disaster epidemiology, post-disaster health monitoring, and community care.
